# Interlayer coupling through a dimensionality-induced magnetic state

**DOI:** 10.1038/ncomms11227

**Published:** 2016-04-15

**Authors:** M. Gibert, M. Viret, P. Zubko, N. Jaouen, J.-M. Tonnerre, A. Torres-Pardo, S. Catalano, A. Gloter, O. Stéphan, J.-M. Triscone

**Affiliations:** 1 Département de Physique de la Matière Quantique, University of Geneva, 24 Quai Ernest-Ansermet, 1211 Genève 4, Switzerland; 2 Service de Physique de l'Etat Condensé, CEA, CNRS, Université Paris-Saclay, CEA Saclay, 91191 Gif-sur-Yvette, France; 3 London Centre for Nanotechnology, University College London, 17-19 Gordon Street, London WC1H 0AH, UK; 4 Department of Physics and Astronomy, University College London, Gower Street, London WC1H 6BT, UK; 5 Synchrotron SOLEIL, L'Orme des Merisiers, Saint-Aubin, BP 48, 91192 Gif-sur-Yvette, France; 6 Institut Néel, CNRS et Université Joseph Fourier, BP 166, 38042 Grenoble, France; 7 Laboratoire de Physique des Solides, Université Paris-Saclay, CNRS-UMR 8502, F-91405 Orsay, France; 8 Departamento de Química Inorgánica, Facultad de Químicas, Universidad Complutense (UCM), CEI Moncloa, 28040 Madrid, Spain

## Abstract

Dimensionality is known to play an important role in many compounds for which ultrathin layers can behave very differently from the bulk. This is especially true for the paramagnetic metal LaNiO_3_, which can become insulating and magnetic when only a few monolayers thick. We show here that an induced antiferromagnetic order can be stabilized in the [111] direction by interfacial coupling to the insulating ferromagnet LaMnO_3_, and used to generate interlayer magnetic coupling of a nature that depends on the exact number of LaNiO_3_ monolayers. For 7-monolayer-thick LaNiO_3_/LaMnO_3_ superlattices, negative and positive exchange bias, as well as antiferromagnetic interlayer coupling are observed in different temperature windows. All three behaviours are explained based on the emergence of a (¼,¼,¼)-wavevector antiferromagnetic structure in LaNiO_3_ and the presence of interface asymmetry with LaMnO_3_. This dimensionality-induced magnetic order can be used to tailor a broad range of magnetic properties in well-designed superlattice-based devices.

## Abstract (web summary)

Oxide materials can be combined to create heterostructures exhibiting complex properties not found in either substance individually. Here, the authors observe antiferromagnetic interlayer exchange coupling between ferromagnetic lanthanum manganite and nominally paramagnetic lanthanum nickel oxide.

## Graphical abstract

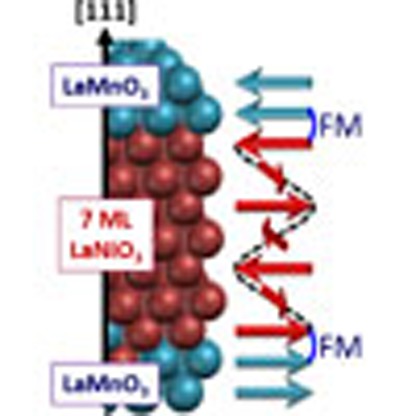

Over the past decade, atomic-scale control of the heterointerfaces between different perovskite oxides has grown to become a well-established route to engineering new functionalities in transition metal oxides^1^,^2^,^3^,^4^. Besides allowing the many exciting properties arising from the collective ordering of their structural and electronic degrees of freedom to be tuned or combined into multifunctional materials, such interfaces can also lead to the emergence of wholly new phases not attainable in bulk. Examples include electron liquids between band insulators or ferromagnetism (FM) at the interface between antiferromagnets (AFs)^5^,^6^,^7^. Strain, charge transfer and electrostatic coupling are just a few of the mechanisms that can be exploited to trigger the new behaviours at transition metal oxide interfaces. One particularly interesting family of oxides is that of the perovskite nickelates *R*NiO_3_, where *R* is a trivalent cation from the lanthanide series^8^,^9^. These compounds are usually insulating AFs at low temperature except for LaNiO_3_ (LNO), which, in its bulk form, is a paramagnetic metal at all temperatures. LNO-based heterostructures have already received considerable attention in the field of interface engineering^10^,^11^,^12^,^13^ and in superlattices made with manganites^14^,^15^,^16^,^17^,^18^,^19^,^20^. In metallic La_2/3_Ba_1/3_MnO_3_/LNO multilayers grown along the (001) direction, interlayer exchange coupling has been reported and interpreted in the frame of RKKY-type interactions through LNO^15^,^21^. Interestingly, it is also known that epitaxial LNO films can display a metal-to-insulator transition as their thickness is reduced to a few unit cells^22^,^23^. In this insulating regime, signatures of antiferromagnetism have been observed^11^.

Here we investigate the properties of ultrathin LNO layers combined with insulating LaMnO_3_ (LMO) in (111)-oriented heterostructures. In its bulk stoichiometric form, LMO is an A-type AF^24^, but when grown as thin film on SrTiO_3_ (STO) substrates, it becomes ferromagnetic below 200 K while still remaining non-metallic, a rare property among ferromagnets^25^,^26^,^27^. The [111] direction used in our superlattices corresponds to the antiferromagnetic propagation vectors of interest in the bulk parent insulating nickelates. Here we report on a unique and complex temperature evolution of the magnetism in these heterostructures. In addition to the negative exchange bias (EB) reported previously^17^, we show that as temperature is increased EB displays a sign reversal before the emergence of an AF-coupled state between the LMO layers. This behaviour occurs exclusively in (111) superlattices with 7-monolayer (ML)-thick LNO and can be explained by the stabilization of an AF spiral with a (¼,¼,¼) wavevector in the ultrathin LNO layers.

## Results

### Structural, magnetic and transport properties

**Figure 1:**
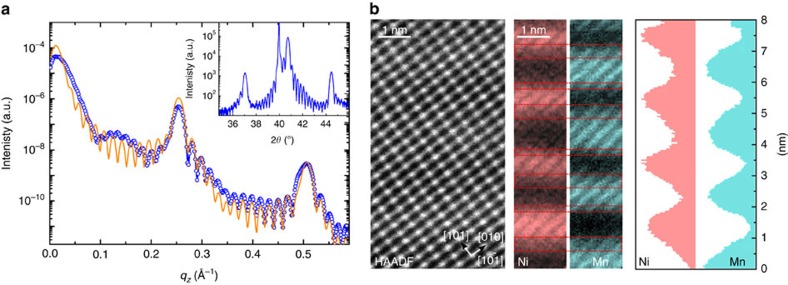
Structural characterization of (111)-oriented LNO/LMO superlattices. (**a**) Reflectivity curve for linear vertical polarization of the light at the Mn L_3_-edge at 300 K (above the Curie point of LMO) for a (LNO_7_/LMO_7_)_15_ heterostructure attesting its excellent structural quality. Dots are experimental measurements and solid lines are fits. Inset: X-ray diffractogram for the same sample. (**b**) High-angle annular dark-field scanning transmission electron microscope images corresponding to a (LNO_5_/LMO_5_)_20_ superlattice projected onto the (10) plane. Growth direction is from bottom to top. Ni and Mn maps and profiles obtained from EELS at the Ni L_3_ (green) and Mn (red) L_2,3_-edges revealing an interfacial structural asymmetry (indicated by rectangles of different width), that is, the LMO-on-LNO interface is more abrupt than the LNO-on-LMO one.

**Figure 2:**
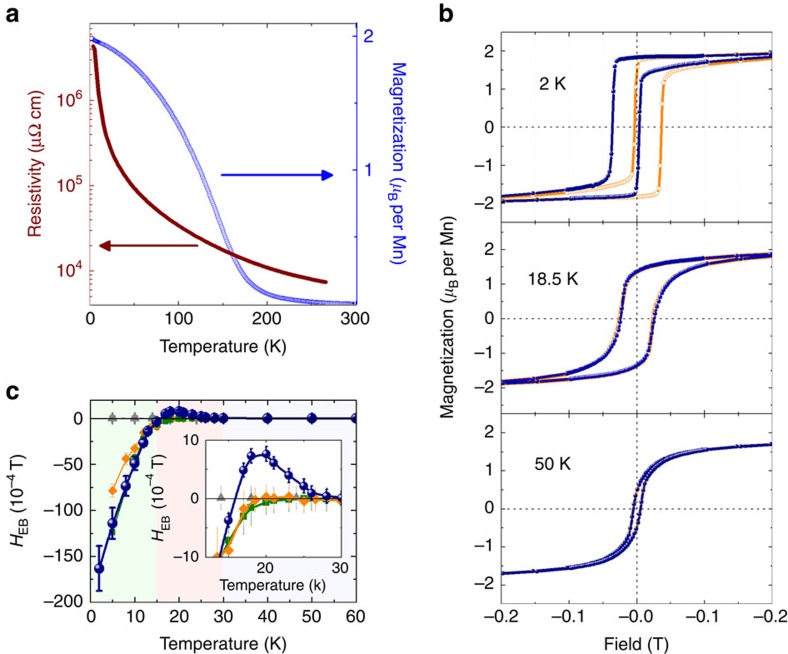
Magnetic and transport properties of (111)-oriented (LNO_7_/LMO_7_)_15_ superlattices. (**a**) Temperature dependence of the resistivity showing a temperature-activated behaviour (left axis) and temperature dependence of the magnetization during field cooling in +0.2 T (right axis). (**b**) Magnetization versus field at 2 (top), 18.5 (middle) and 50 K (bottom) after a field-cooling process in +0.2 T (closed blue symbols) and −0.2 T (open orange symbols). The rounding of the 50 K loop hints at the existence of antiferromagnetic interactions. (**c**) Summary of the EB field *H*_EB_ after field cooling in +0.2 T as a function of temperature showing that the *H*_EB_ changes sign above 15 K (*H*_EB_>0) before vanishing at ∼30 K (*H*_EB_=0). Different background colours indicate the different EB regions. The evolution of *H*_EB_ with temperature is also shown for the (111) superlattices (LNO_5_/LMO_7_)_17_ (green) and (LNO_8_/LMO_7_)_14_ (orange) and for a 57-ML-thick (111)-LMO (grey) thin film. EB sign reversal is exclusively observed for the superlattice with *N*=7 MLs.

[(LNO)_*N*_/(LMO)_*M*_]_*X*_ superlattices, where *N* and *M* are the number of MLs in each layer—the metal–metal distance in the (111) direction—and *X* the number of repetitions of the stack, were grown on (111)-oriented STO substrates. As shown in Fig. 1a, strong superlattice peaks and thickness fringes are clearly observed in X-ray reflectivity, and diffraction measurements demonstrating the high quality of the (111)-oriented heterostructures investigated. The coherent epitaxial growth and the absence of secondary phases or dislocations are confirmed by high-resolution high-angle annular dark-field scanning transmission electron microscopy and electron energy loss spectroscopy (EELS; Fig. 1b). Interestingly, throughout the superlattice structure, the two interfaces are found not to be structurally equivalent: when LMO is deposited on LNO, a very sharp interface is obtained (roughness of one ML), whereas the LNO-on-LMO interface is intermixed on the scale of two to three MLs (Fig. 1b, right panels)^28^. Moreover, reducing the LNO thickness results in the loss of the metallic character observed in the thicker layers as can be seen in Fig. 2a, where the resistivity of the (LNO_7_/LMO_7_)_15_ heterostructure displays a temperature-activated dependence in this low LNO thickness regime (*t*_7LNO-[111]_∼1.5 nm). This confirms that the dimensionality-induced insulating character of LNO, previously reported in (001)-oriented structures, is also observed in (111)-LNO/LMO superlattices. Figure 2a also shows the temperature dependence of the magnetization for a (LNO_7_/LMO_7_)_15_ superlattice after cooling in +0.2 T, whereas magnetization-field loops at different temperatures, acquired after both positive and negative field-cooling processes, are displayed in Fig. 2b. At 2 K, square hysteresis loops shifted along the field axis are observed, consistent with the presence of the previously reported negative EB^17^. Surprisingly, the EB changes sign at ∼15 K, before disappearing at ∼30 K to give way to rounded magnetic loops with remnant magnetization that decreases rapidly with temperature (Supplementary Fig. 1). The latter behaviour hints at some degree of AF coupling between the LMO layers in the heterostructures. Figure 2c summarizes the temperature evolution of the EB field *H*_EB_, defined as the offset of the hysteresis loop along the field axis. It is worth noting that whereas negative EB occurs at low temperature for all periodicities, EB sign reversal is exclusively observed for the (LNO_7_/LMO_7_)_15_ superlattices. Successive magnetic training measurements (shown in Supplementary Fig. 2) proved that the positive EB is an intrinsic effect of the heterostructure and cannot be attributed to disorder^29^.

### Interlayer coupling between LMO layers

**Figure 3:**
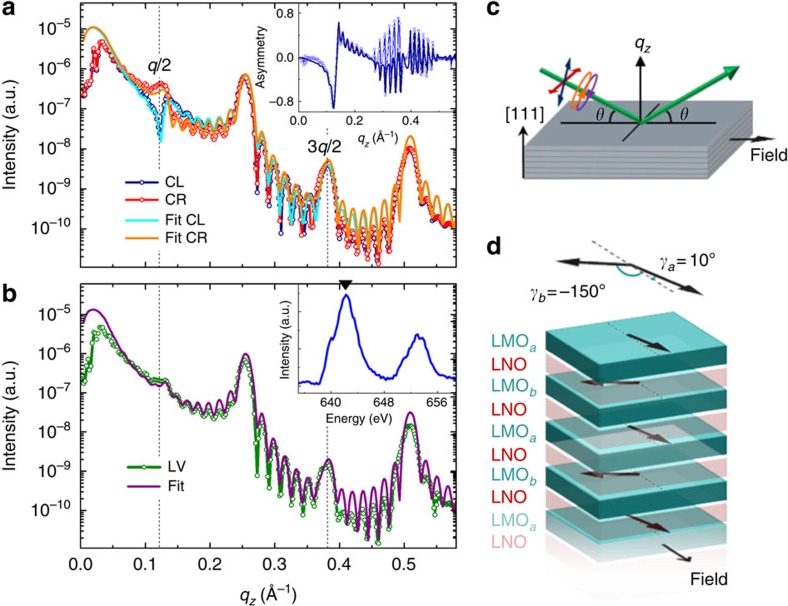
Soft X-ray reflectivity at the Mn L_3_-edge of the (LNO_7_/LMO_7_)_15_ superlattice at 30 K and in 0.05 T after cooling in the same field. (**a**) Reflectivities for circularly left (CL, blue line) and right (CR, red line) polarized light. Inset: extracted asymmetry ratio (CR−CL)/(CR+CL). (**b**) Reflectivity with linearly vertical (LV) polarized light. For simplicity, linear horizontal polarization is not shown. In all reflectivity curves, points are experimental measurements and solid lines are fits. Inset: corresponding Mn L_2,3_ X-ray absorption spectra. The arrow in the lower inset indicates the energy at which reflectivity measurements were performed. (**c**) Schematics of the scattering geometry for reflectivity measurements. (**d**) Sketch of the extracted magnetic configuration showing that the doubling of the magnetic structure along the normal direction corresponds to two LMO sublattices with their net magnetic moments oriented at 160° from each other.

To investigate the nature of the magnetic state in the regime where EB has vanished (*T*≥30 K), we performed polarization-dependent resonant X-ray reflectivity measurements at Mn L_2,3_-edges. This technique is particularly well-suited to unveiling the depth-resolved magnetic profile of such heterostructures. Figure 3a,b presents the reflectivity spectra measured on a (LNO_7_/LMO_7_)_15_ superlattice at the Mn L_3_-edge (642.5 eV) at 30 K after field cooling in 0.05 T. Reflectivity curves were acquired both with circular left and right, as well as with linear vertical and horizontal polarizations in specular geometry with the magnetic field applied parallel to the intersection between the sample surface and the scattering plane (Fig. 3c). Compared with the corresponding room temperature measurements (Fig. 1a), the emergence of ½-order peaks at *q*/2 and 3*q*/2 is clearly visible in these low-temperature spectra. The positions of these resonant peaks correspond to a real space doubling of the superlattice periodicity, providing evidence for the existence of two magnetically different LMO layers. The analysis of the full set of measurements obtained with both circularly (Fig. 3a) and linearly (Fig. 3b) polarized light allows the directions of individual LMO sublattice magnetizations to be determined and their canted antiferromagnetic arrangement to be confirmed. The structural parameters were extracted from fits to the reflectivity curves at 300 K (Fig. 1a), that is, above the Curie point. Keeping the number of free parameters as small as possible, layer thicknesses of LNO=1.31 nm and LMO=1.16 nm were obtained, in fair agreement with the nominal values, along with a typical interface intermixing of 0.4 nm (assumed constant throughout heterostructure for simplicity of the fits). A magnetization value of 2.3 μ_B_ per Mn was also inferred from SQUID-magnetometry at saturation and single LMO layers. Keeping all these parameters constant, the entire set of low-temperature curves obtained with circularly and linearly polarized X-rays were fitted using only the two independent angles of the magnetization sublattices with respect to the applied field direction as free parameters. The best fits for the (7/7) period multilayer (Fig. 3a,b) reveal that when field cooled to ∼30 K in 0.05 T the two LMO sublattice magnetizations are oriented, respectively, at 10° and −150° from the direction of the applied magnetic field, thus making an angle of 160° between them (Fig. 3d). The inset in Fig. 3a shows the agreement for the magnetic asymmetry, which is very sensitive to the orientation of the magnetic moment within the alternating layers. Fits assuming an antiparallel alignment between the two LMO sublattices rendered a total magnetization value much lower than the one extracted from SQUID-magnetometry. Further resonant magnetic reflectivity measurements showed that when a larger magnetic field is applied, the magnetizations of the two LMO sublattices fold progressively and end up parallel near 0.3 T (this is shown in Supplementary Fig. 3). A significant coupling energy of 0.3 mJ m^−2^ can then be inferred straightforwardly by considering that the Zeeman energy at that field compensates the interlayer coupling. Interestingly, the AF arrangement of the LMO layers is only obtained for the (111)-oriented superlattices with *N*=7 MLs and drops markedly once the LNO layer thickness departs from this value; that is, superlattices with LNO thicknesses of *N*≠7 do not show the ½-order peaks (Supplementary Fig. 4). As discussed below, the fact that the 7-ML-LNO thickness is a very special case is a central clue for a possible explanation of the coupling behaviour through the (111)-LNO layers.

### Magnetism in LNO

**Figure 4:**
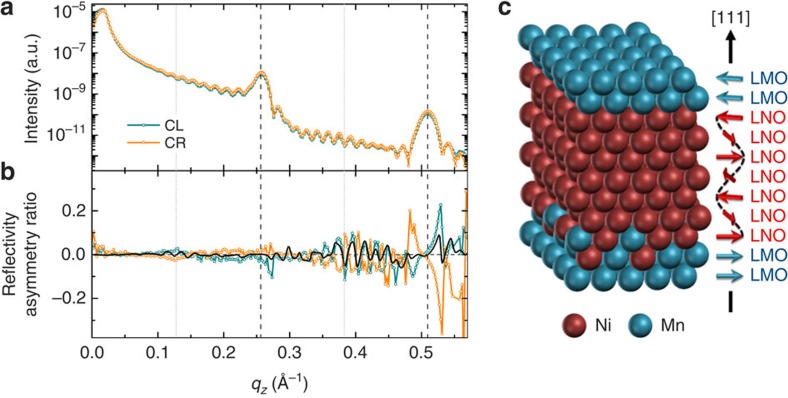
Soft X-ray reflectivity at the Ni L_2_-edge for the (LNO_7_/LMO_7_)_15_ superlattice at 30 K after cooling in 0.05 T. (**a**) Reflectivities for circularly left (CL, blue line) and right (CR, orange line) polarized light acquired in 0.05 T. (**b**) Reflectivity anisotropy ratio ([*I*(*H*)−*I*(−*H*)]/[*I*(*H*)+*I*(−*H*)]), obtained by reversing the sign of the 0.1 T field between each angular step, for CL (blue dots) and CR (orange dots) polarized light, and the corresponding fit to the model for the CL polarization (solid black line). Vertical lines indicate ½-, first-, and second-order Bragg peaks. (**c**) Sketch of the proposed magnetic arrangement in (111)-LNO. The (¼,¼,¼) order is stabilized by the ferromagnetic coupling with the LMO on both sides, and the resulting interaction between neighbouring LMO layers through 7 MLs of LNO is antiferromagnetic.

As previously mentioned, bulk LNO is a paramagnetic metal but it can acquire some magnetic properties when ultrathin insulating layers are sandwiched in a heterostructure configuration^11^,^17^,^18^,^19^,^30^,^31^,^32^,^33^,^34^. Specifically, a non-collinear AF order with a (¼,¼,¼) pseudocubic wave vector analogous to the one displayed by all other members of the perovskite nickelates family has been measured for LaAlO_3_/LNO superlattices grown along the (001) direction^11^,^30^. We suggest here that such a 4-unit-cell-period magnetic superstructure along the [111]_pc_ direction is at the origin of the AF coupling observed in our (111)-oriented LNO/LMO superlattices with *N*=7, since this particular (111)-LNO thickness would specifically favour an AF arrangement between the LMO layers as illustrated in Fig. 4c. Within this model, a LNO thickness of 3 and 11 MLs should also lead to AF coupling between LMO layers. Unfortunately, *N*=3 superlattices are not smooth enough, whereas the *N*=11 ones are metallic and do not seem to stabilize the (¼,¼,¼) antiferromagnetic structure. For the other (111)-LNO thicknesses, the coupling is expected to be either ferromagnetic or at 90°. In the latter case, any change of chirality of the (¼,¼,¼) spin arrangements would actually modify the coupling from 90° to −90°. The resulting randomness would favour a multidomain state in the LMO layers, or at least incomplete local magnetization. This behaviour is in agreement with both magnetometry and reflectivity measurements of superlattices with *N*≠7, where no ½-order peaks have been observed (as shown in Supplementary Fig. 4). The behaviour of the 7-ML-LNO superlattices has been checked in three different samples. The insulating character of the very thin LNO layers, along with the ferromagnetic coupling to LMO at each interface^18^,^19^,^35^, contribute to stabilize the (¼,¼,¼)-magnetic superstructure, otherwise fluctuating in metallic LNO^31^,^36^. The existence of an interface-induced moment in Ni, coupled parallel to the LMO magnetization, was indeed inferred from X-ray magnetic circular dichroism measurements in several (LNO/LMO) multilayers^35^, as also found in other published works^18^,^19^. This is expected through the effect of ferromagnetic superexchange between Ni^2+^ and Mn^4+^, that is, in the presence of interfacial charge transfer between LMO and LNO, and reinforced by the slight intermixing at interfaces. In both cases the local magnetic properties would be close to those of the ordered La_2_MnNiO_6_ compound^37^, that is, a ferromagnetic alignment of Mn and Ni spins.

To look for a signature of the proposed antiferromagnetic structure in LNO, we also performed X-ray reflectivity measurements at the Ni L_3,2_-edge. Unfortunately, the Ni L_3_ transition is dominated by the contribution from the La M_4_-edge forcing us to carry out the measurements at the less intense Ni L_2_-edge (870.75 eV). As a result, the magnetic contribution to the reflectivity curves is not as clear as those at the Mn edge, and no obvious magnetic Bragg peak could be seen at a position corresponding to the (¼,¼,¼) structure (Supplementary Fig. 5). However, this is not a surprise given that its amplitude is weak compared with the Kiessig fringes and the 7-ML-LMO spacers prevent a full coherence of this structure through the entire superlattice thickness, resulting in a drastically reduced Bragg peak intensity (as shown in Supplementary Fig. 6). Nevertheless, a small but clear asymmetry can be observed on reversal of the 0.1 T field in measurements carried out with circular right and circular left polarizations, as shown in Fig. 4b. The mirror effect observed in these measurements attests the magnetic origin of the data. Four main features (indicated by the vertical lines in Fig. 4b) can be distinguished around the positions of the first- and second-order Bragg peaks, as well as at the ½- and -order peaks, evidencing some degree of antiferromagnetic order between adjacent LNO layers. To fit the data, each LNO layer was decomposed into three parts: two interfacial layers with magnetization parallel to that of the neighbouring LMO layer (the angle between the two LMO sublattice magnetizations being 160°) and an average central magnetization that is free to rotate. To obtain good agreement between the fit and the data of Fig. 4b, a 1-Å thickness variation in the individual layers along the superlattice thickness had to be introduced, thereby increasing the complexity of the model. Nevertheless, the most obvious feature located at *q*_*z*_∼0.53 Å^−1^, close to the second multilayer Bragg peak, cannot be accounted for by any model assuming collinear magnetization in Ni (for example, homogeneous magnetization or a decaying magnetic profile). Thus, the important result from the fitting is that the magnetization of the inner parts of the LNO layers is found to be mainly perpendicular to that of the interfaces. This result is fully consistent with the proposed (¼,¼,¼) magnetic structure in LNO, which would generate a global uncompensated perpendicular magnetization component at the centre of the LNO layers.

### Evolution of exchange bias in 7-ML-thick-LNO superlattices

**Figure 5:**
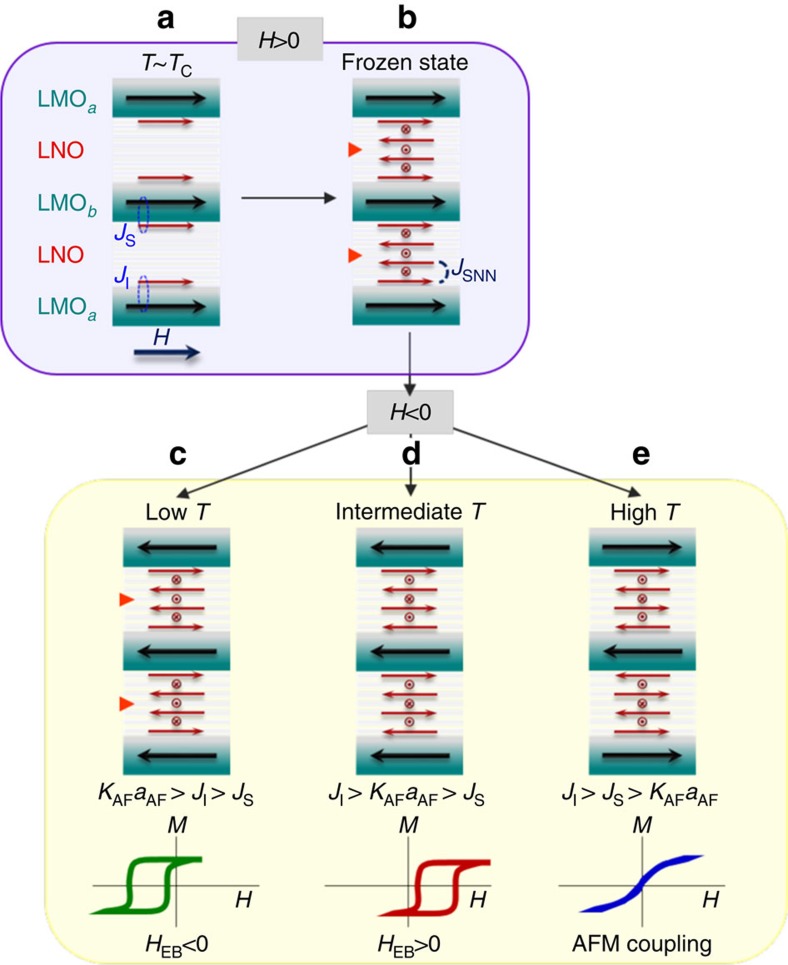
Exchange bias evolution for a superlattice with 7-ML-thick (111)-LNO layers. Schematics of the field-cooling procedure: (**a**) at the FM ordering temperature, the LMO layers induce a moment in the interfacial Ni, which (**b**) subsequently stabilizes a magnetic defect (orange triangles) in the (¼,¼,¼) AF order. This configuration freezes in during field cooling and gives the starting point for the field measurements. (**c**) At low temperature, the anisotropy in LNO is large and the magnetic defect is frozen inside these layers. At negative field, both interfacial exchange energies *J*_S_ and *J*_I_ are frustrated, resulting in the existence of negative EB. As temperature increases, the anisotropy weakens and becomes smaller than the larger of the two interface exchanges (*J*_I_). (**d**) In this intermediate-temperature case, a negative field reverses the Ni spins on one side of the LMO interface and annihilates the magnetic defect in LNO. This configuration is stabilized if *J*_S_ is the smallest energy scale, thus inducing a sign change of the exchange bias field. (**e**) At higher temperature, the anisotropy is negligible and all the energy terms are minimized when the LMO layers are AF-ordered.

The overall coupling between neighbouring LMO layers mediated by 7 MLs of the LNO AF structure is thus antiferromagnetic, as schematized in Fig. 4c. This coupling is only possible along the [111] direction and for a LNO thickness of 7 MLs, in agreement with our data. Considering such a coupling through LNO, the challenge now is to explain the magnetic properties of the (LNO_7_/LMO_7_)_15_ superlattices in the entire temperature range, including the EB and its sign change—sign change that is only observed for (LNO_7_/LMO_7_)_15_ superlattices as shown in Fig. 2c. It is known that several magnetic interactions are at play in conventional FM/AF exchange-biased systems^38^, comprising the resulting magnetic ordering of the layers and their interface coupling. Interestingly, in our superlattices, transmission electron microscopy measurements indicate that the LNO/LMO and LMO/LNO interfaces are not equivalent as can be seen in Fig. 1b (ref. 28). In the present case, X-ray absorption spectroscopy (XAS) and EELS measurements performed on LMO/LNO heterostructures show that charge transfer is larger for the more intermixed interface^28^, which will likely unbalance the strength of interfacial coupling on both sides of the ferromagnetic layer. Indeed, while intermixing leads to an alloy where strong Mn^4+^/Ni^2+^ FM superexchange should dominate (as in the double perovskite La_2_MnNiO_6_), the smoother interface should give rise to competing AF contributions from Mn^3+^/Ni^3+^ superexchange. Thus, while still FM-coupled, the sharp interface should lead to a smaller exchange (*J*_S_) than the more intermixed LNO-on-LMO one (*J*_I_): *J*_I_>*J*_S_. In addition, there are two other relevant energy scales linked to the antiferromagnetic LNO structure. The first one is the single-atom anisotropy, *K*_AF_, and the second the energy of a planar AF defect, which is of the order of the second nearest-neighbour exchange in LNO, *J*_SNN_. Like in most conventional exchange-biased systems, the AF anisotropy and exchange are the quantities that vary most with temperature and are responsible for the ‘freezing' of the AF state below the blocking temperature. Thus, one can imagine that *K*_AF_ goes from negligible at high temperatures to values larger than the interface exchanges at low temperature. During the field-cooling procedure, a likely scenario is depicted on the top part of Fig. 5. At high temperature, LMO becomes magnetic and drives the interfacial Ni moments to align with those of LMO (Fig. 5a), but the (¼,¼,¼) structure is not yet stable in LNO. Once it stabilizes, it has to adapt to the parallel LMO/LNO interfaces, which impose a magnetic phase shift in the 7-ML LNO. This would generate a magnetic defect in the LNO layer, as sketched by the orange triangles in Fig. 5b, which costs an energy of the order of *J*_SNN_. When the temperature decreases, this structure freezes in as the anisotropy of the Ni moments closer to the interface establishes a potential energy barrier preventing the magnetic defect from moving. At very low temperatures (Fig. 5c), reversing the magnetization of the ferromagnetic LMO layers does not affect the frozen AF-LNO configuration, and the total energy increases through the additional frustration of the two interface couplings. This produces EB with the classic negative sign shift (*H*_EB_<0) of the hysteresis cycle. This scenario has common points with the models of Mauri *et al*.^39^ and Kiwi^38^ for conventional exchange bias where an AF planar domain wall is wound in the AF. The main difference here is that the particular AF structure of LNO is likely to allow for a magnetic phase slip on a single-unit-cell scale. As the temperature is raised (Fig. 5d), the AF anisotropy decreases below the larger interface exchange energy *J*_I_ (but still above *J*_S_). At this interface, the strong *J*_I_ locks the interfacial Ni spins and forces them to follow the Mn magnetization, at the (lower) cost of some anisotropy energy. When the LMO magnetization reverses, the rotation of Ni moments annihilates the AF defect. The total energy of this final state is decreased if *J*_S_<*J*_SNN_, in which case the EB changes sign (*H*_EB_>0). The observed sign reversal is noteworthy as reports of positive EB are scarce and its observation usually requires a different cooling procedure under a much larger field (for example, the FeF_2_/Fe system)^40^. Here the sign change results from a temperature-induced crossing of anisotropy energy with one (and only one) of the interfaces' exchanges. At higher temperature (Fig. 5e), the anisotropy decreases further and the Ni moments at both interfaces become locked to those of Mn. The EB therefore disappears and the system can be considered to be above the blocking temperature. In this case, the most stable state is the one where no interaction is frustrated, that is, the AF-coupled LMO layers, and this is indeed what is unambiguously observed in the synchrotron reflectivity measurements.

We would like to stress here the fundamental difference between the present dimensionality-induced interlayer coupling and the previously proposed RKKY mechanism in metallic (001) superlattices^21^,^33^. In the present case, the non-metallic nature of our system precludes a coupling mediated by conduction electrons^41^,^42^ with the formation of standing waves in the interlayer resulting from spin-dependent electron interface reflectivity. Although this mechanism is, in principle, also possible in insulators where Bloch states in the spacer are replaced by evanescent states exponentially decaying with distance from the interfaces^42^,^43^, it leads to a reduced coupling strength that increases with temperature, in qualitative disagreement with our temperature-dependent measurements (not shown here). Moreover, the insulating nature of our ferromagnetic LMO layers drastically reduces the density of evanescent states, making a coupling mediated by conduction electrons very unlikely. Finally, the strong LNO thickness dependence observed (adding or subtracting one LNO ML eliminates the AF exchange) provides further evidence for the digital nature of the magnetic coupling consistent with the emergence of the 4-unit-cell AF structure along the [111] direction in LNO.

The complex magnetic behaviour observed in (111)-oriented LNO/LMO superlattices can thus be attributed to a cascade of phenomena, which arise from the conjunction of two main factors: reduced dimensionality and interface asymmetry. The former leads to the loss of metallic character and the appearance of non-collinear AF order with a (¼,¼,¼)-propagation vector in the ultrathin (111)-LNO confined between insulating LMO layers. The stabilization of the AF structure, together with the ferromagnetic superexchange between the Ni and Mn ions, provide the basic ingredients for both the development of EB and the long-distance interaction responsible for the unusual coupling between the neighbouring LMO layers. The 4-unit-cell periodicity of the AF structure along the [111] direction in LNO is key to understanding the strong thickness dependence of the observed interlayer coupling. The effect of the interface asymmetry, resulting from different degrees of intermixing, is to break the equivalence of two interfacial exchanges. This in turn leads to a non-trivial temperature dependence of the exchange bias as the atomic exchange energy sequentially crosses the two interfacial exchanges on cooling. The complexity of the magnetic behaviour observed in this seemingly simple, two-component system illustrates the great potential that oxide heterostructures hold for engineering new functionalities and tailored magnetic properties.

## Methods

### Sample fabrication and structural characterization

[(LNO)_*N*_/(LMO)_*M*_]_X_ superlattices were grown on (111)-oriented STO substrates by radiofrequency off-axis magnetron sputtering at 510–600 °C in 0.18 mbar of Ar:O_2_ mixture of ratio 3:1 (ref. 17). Note that the thickness of a ML corresponds to the metal–metal distance along the [111] direction, which is shorter than that along the most common (001) growth direction. X-ray diffraction scans were obtained with a high-resolution PANalytical X'Pert PRO diffractometer using the Cu Kα_1_ radiation.

### Magnetic and transport measurements

Magnetic properties were measured in a Quantum Design VSM system in the temperature range 2–300 K, with the magnetic field applied in the plane of the sample. The error bars of the EB field *H*_EB_ were determined from the uncertainty in the values of the coercive fields due to the limited number of data points in the magnetization-field loops. The electric measurements were performed in a standard 4-probe configuration in the temperature range 4–300 K after appropriate patterning of the samples.

### Transmission electron microscopy

Cross-sections of the (LNO/LMO)//(111)-STO heterostructures were prepared by first mechanically polishing the structures using the tripod method and then further thinning to electron transparency with a precision ion-polishing system. High-resolution high-angle annular dark-field scanning transmission electron microscopy and EELS were performed using a NION200 aberration-corrected microscope.

### X-ray resonant reflectivity

X-ray resonant reflectivity measurements were performed at the SEXTANTS beamline at the synchrotron SOLEIL, France^44^. The reflectivity curves were acquired with circularly and linearly polarized light with the energy tuned to the Ni and Mn L-edges. Measurements were performed at 300 and 30 K with the magnetic field applied parallel to the plane of the sample during the cooling process. Several different samples were measured to check the reproducibility of the results. The simulations were carried out using the DYNA software^45^, with the optical constants derived from X-ray magnetic circular dichroism measurements at the Mn L_2,3_-edge previously performed for equivalent superlattices^35^. To minimize the number of free parameters for the magnetic spectra, we reduced the number of structural parameters such as to fit primarily the charge peaks (that is, positions, relative intensities and shape).

## Additional information

**How to cite this article:** Gibert, M. *et al*. Interlayer coupling through a dimensionality-induced magnetic state. *Nat. Commun.* 7:11227 doi: 10.1038/ncomms11227 (2016).

## Supplementary Material

Supplementary Information Supplementary Figures 1-6 and Supplementary Notes 1-2.
