# Supplementary material for: Interlayer coupling through a dimensionality-induced magnetic state
Source: Nat Commun. 2016 Apr 15;7:11227. doi: 10.1038/ncomms11227 (PMC4835538; doi:10.1038/ncomms11227)
Supplement: Supplementary Information — Supplementary Figures 1-6 and Supplementary Notes 1-2. [file ncomms11227-s1.pdf]

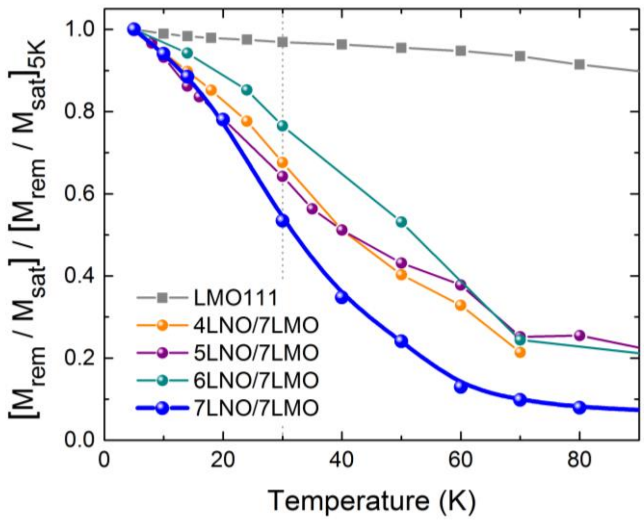

Supplementary Figure 1: Temperature-dependence of the ratio between remnant magnetization and saturation magnetization (*M_{rem}/M_{sat}*) for (111)-oriented LNO/LMO superlattices normalized with their values at 5 K. This parameter quantifies the squareness of hysteresis curves for the superlattices with periodicities: $(LNO_4/LMO_7)_{17}$ (orange), $(LNO_5/LMO_7)_{17}$ (purple), $(LNO_6/LMO_7)_{16}$ (cyan) and $(LNO_7/LMO_7)_{15}$ (blue). A 57-monolayer-thick LMO thin film grown on (111)STO (grey) is also shown for comparison. All measurements were acquired after a field cooling process in +0.05 T. The vertical dashed line indicates the temperature at which the antiferromagnetic coupling between the LMO layers is observed for the $(LNO_7/LMO_7)_{15}$ superlattice.

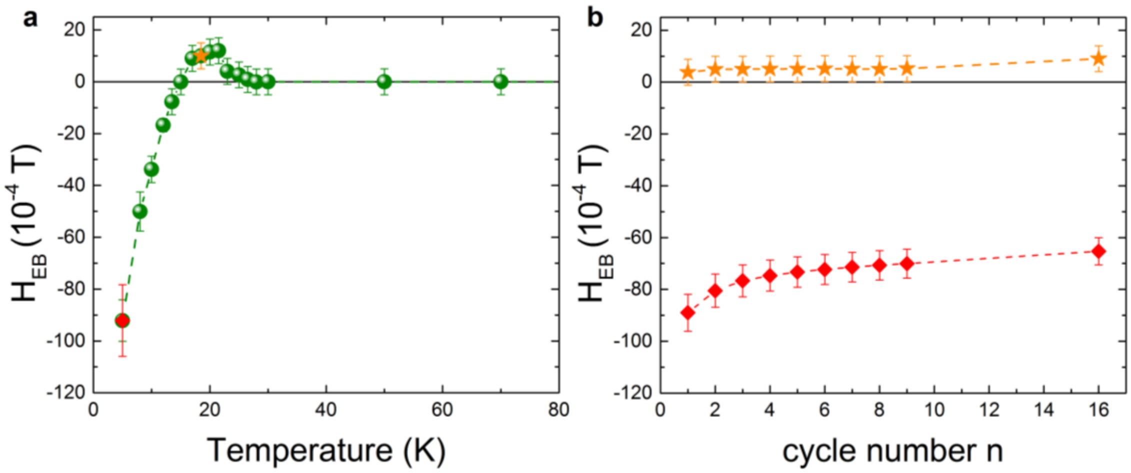

Supplementary Figure 2: Training effect in a (LNO_7/LMO_7)$_{15}$ superlattice. (**a**) Exchange bias field H_{EB} as function of temperature. Red and orange symbols indicate the temperatures at which training effects have been investigated. (**b**) Evolution of the exchange bias field H_{EB} with number of cycles at 5 K (red) and 18.5 K (orange). *M(H)* loops were acquired after a field cooling process in +1 T. The error bars of the *EB* field H_{EB} were determined from the uncertainty in the values of the coercive fields due to the limited number of data points in the magnetization–field loops

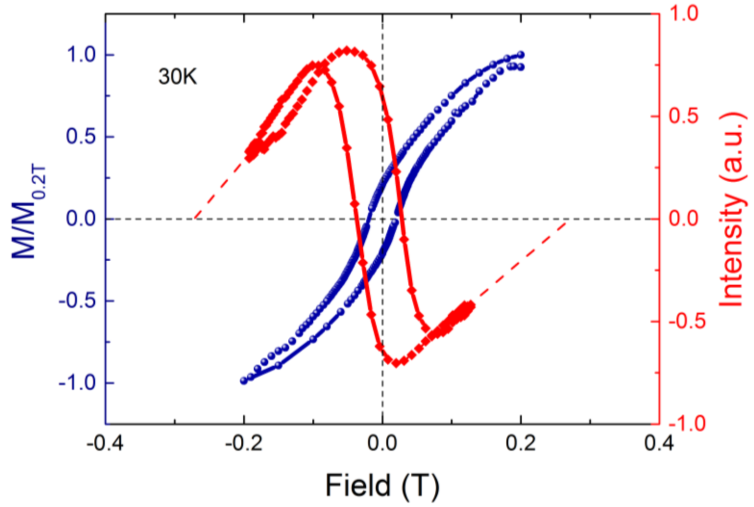

Supplementary Figure 3: Field dependence of the magnetization of the $(LNO_7/LMO_7)_{15}$ superlattice from SQUID measurements (blue, left axis) and concomitant intensity of the q/2 peak at Mn L_3-edge (red, right axis). Both measurements were acquired after field cooling in 0.05 T to 30 K. Red dashed lines extrapolate the saturation filed.

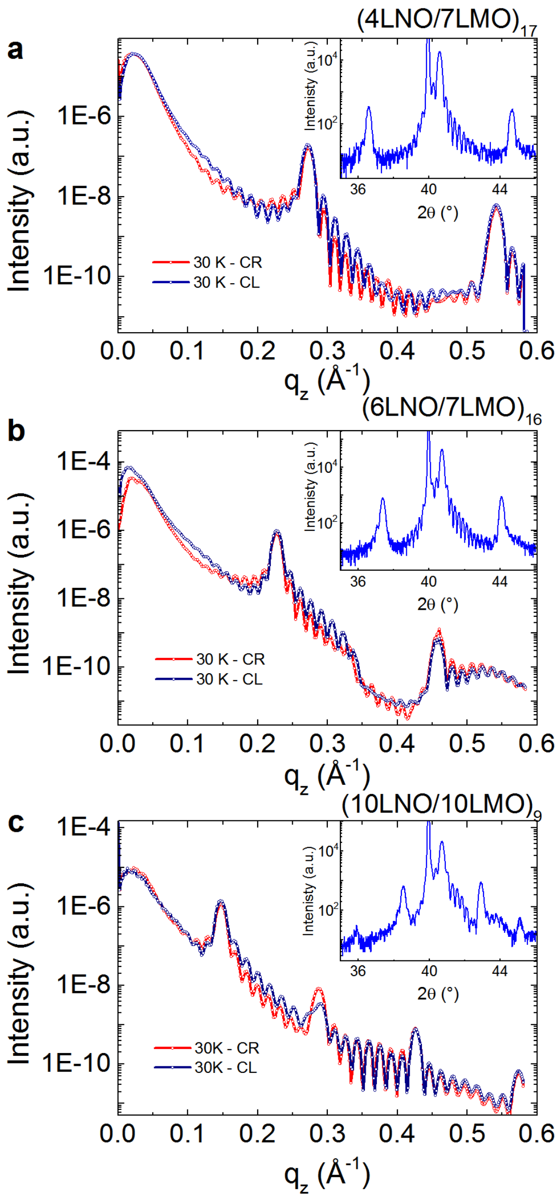

Supplementary Figure 4: X-ray reflectivity at the Mn L_3-edge in circularly left (CL, blue line) and right (CR, red line) polarised light at 30 K and in 0.05 T, after cooling in the same field. (**a**) $(LNO_4/LMO_7)_{17}$//STO(111), (**b**) $(LNO_6/LMO_7)_{16}$//STO(111) and (**c**) $(LNO_{10}/LMO_{10})_9$ //STO(111). Insets: X-ray diffractogram for the corresponding superlattices.

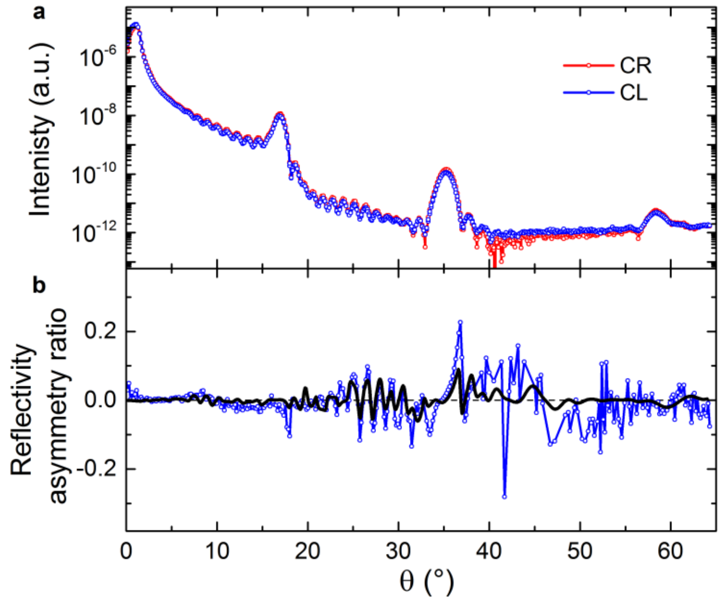

Supplementary Figure 5: Soft X-ray reflectivity measurements at the Ni L_2-edge of the (111)-oriented $(LNO_7/LMO_7)_{15}$ superlattice at 30 K after cooling in 0.05 T. (**a**) Reflectivity curves acquired in CL (blue line) and CR (red light) polarised light at 0.05 T. (**b**) Reflectivity anisotropy ratio ([*I*(*H*)-*I*(-*H*)]/[*I*(*H*)+*I*(-*H*)]) in a 0.1 T field reversed for each angular step in CL (blue dots) and corresponding fit (black line).

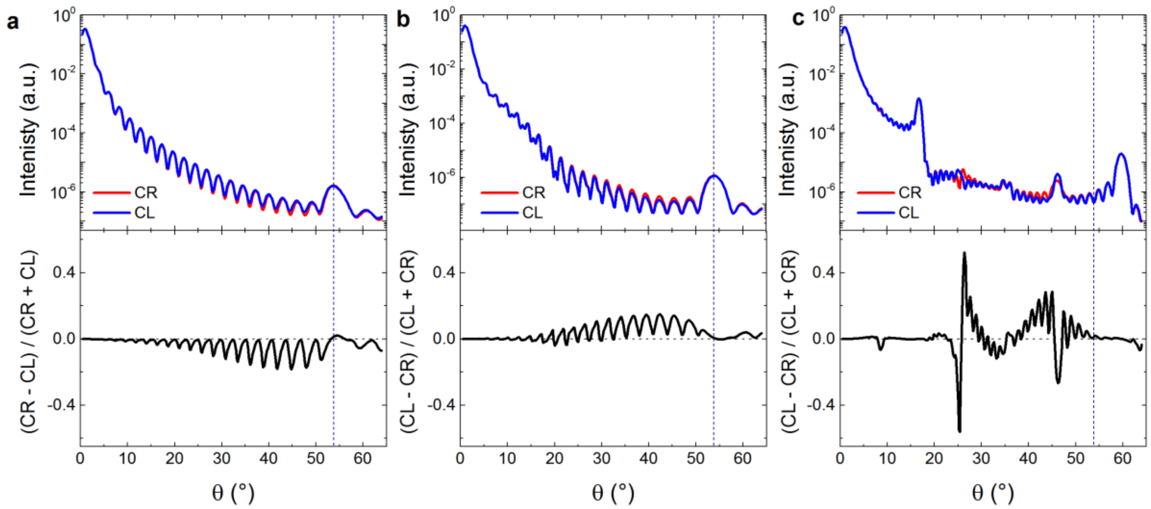

Supplementary Figure 6: Simulations of the X-ray reflectivity curves (top panel) and asymmetry ratio (CR-CL)/(CR+CL) (bottom panel) at the Ni L_3-edge. (**a**) (111)-oriented 98-monolayer-thick LNO film with a spiral antiferromagnetic order. The expected (¼ ¼ ¼)-peak is distinguished around ~54° (dashed vertical line), adding to the structural scattering. (**b**) (111)-oriented 98-monolayer-thick LNO film with a (¼ ¼ ¼)-antiferromagnetic order plus an additional LMO layer on top in order to get the same thickness of the LNO/LMO superlattice measured. (**c**) Perfect (111)-oriented $(LNO_7/LMO_7)_{15}$ superlattice with antiferromagnetic spirals in each LNO layer. With this real stack, the structural scattering associated with the different periods completely takes over. The (¼ ¼ ¼)-peak is just vaguely distinguished, both in the reflectivity curves and in the asymmetry ratio. This demonstrates that a clear Bragg peak cannot be expected in our superlattices, since the structural intensity in reflectivity geometry is large and it amplifies the magnetic components at the *q* values corresponding to structural periods.

Supplementary Note 1: SQUID-magnetometry measurements

Supplementary Figure 1 presents the temperature-evolution of the squareness of the magnetization-field loops for several (111)-oriented $LaNiO_3/LaMnO_3$ (LNO/LMO) heterostructures, as represented by the ratio between remnant (M_{rem}) and saturation (M_{sat}) magnetizations. The superlattice with 7-monolayer-thick-LNO is singled out as the one clearly displaying the fastest decay of the M_{rem}/M_{sat} ratio with temperature. Such behavior is a strong indication of the increasing strength of the interlayer antiferromagnetic coupling above 30 K, as already evidenced in the resonant reflectivity measurements. It is also consistent with a strengthening of the global magnetic behaviour at lower temperature due to the exchange bias field. The decrease of M_{rem}/M_{sat} with temperature observed for the superlattices compared to a bare LMO film (also shown in Supplementary Figure 1) is explained by a reduction of the LMO magnetization and ordering parameter at the interface.

Training effects have been investigated by cycling the system through several consecutive hysteresis loops at a given temperature. Supplementary Figure 2 reports the magnetization-field loops for a $(LNO_7/LMO_7)_{15}$ superlattice acquired at T=5 K and T=18.5 K, two temperatures representative of the negative and positive biasing regimes, respectively. The exchange bias sign does not change upon cycling, which demonstrates that the positive exchange bias is an intrinsic effect of our LNO/LMO heterostructures.

Supplementary Note 2: Reflectivity measurements at Mn L_2-edge

Supplementary Figure 3 presents the field-dependence of the half-order peak $q/2$ indicative of an antiferromagnetically-coupled state in $(LNO_7/LMO_7)_{15}$ superlattices. Its behaviour is consistent with the SQUID measurements that show a low remanence and a rather slow saturation in field. When sweeping the field from saturation, antiferromagnetic coupling takes over leading to a maximum intensity between 0.05 and 0.1 T (depending on the sweep direction). Then, upon reversal of the field, the entire magnetic structure flips and produces a minimum in the $q/2$ peak intensity. The asymmetric positions of the field of these maximum and minimum are attributed to the tilt of the small ferromagnetic component. The two independent measurements of the $q/2$ peak asymmetry ratio and the net magnetization at each field step allow the 'high' field induced folding of the magnetization vectors to be reconstructed. This is represented in Supplementary Figure 3 where saturation can be expected to occur near 0.3 T from a rough extrapolation of the linear and reversible part. The evolution of the $3q/2$ peak intensity as function of temperature suggests that the antiferromagnetic-coupled state vanishes around 120 K.

The absence of half-order peaks in reflectivity measurements at Mn L_3-edge for superlattices with LNO thickness $N\neq7$ monolayers is evidenced in Supplementary Figure 4.
